# Optimized electrical and physiochemical properties of cadmium telluride thin films via copper chloride treatment for photovoltaic applications

**DOI:** 10.1038/s41598-026-36991-4

**Published:** 2026-02-12

**Authors:** Camellia Doroody, Muhammad Najib Harif, Zheng-Jie Feng, Armin Rajabi, Mohammad Yeganeh Ghotbi, Fazliyana ‘Izzati Za’abar, Allina Binti Nadzri, Kazi Sajedur Rahman, Yasser Fouad, Chenyoushi Xu, Manzoore Elahi M. Soudagar, Mahaboob Patel

**Affiliations:** 1 School of Engineering, Lishui University, Lishui, 323000 Zhejiang China; 2 Zhejiang Xingyu Mechanical and Electrical Technology Company Ltd, Tongqin Town, Wuyi County, Jinhua, 311400 Zhejiang China; 3 Centre for Promotion of Research, Graphic Era (Deemed to be University), Clement Town, Dehradun, India; 4 Faculty of Applied Sciences, Universiti Teknologi MARA, Cawangan Negeri Sembilan, Kuala Pilah, Malaysia; 5 Institute of Sustainable Energy, Universiti Tenaga Nasional (@The Energy University), Jalan IKRAM-UNITEN, 43000 Kajang, Selangor Malaysia; 6 Institute of Power Engineering, Universiti Tenaga Nasional (@The Energy University), Jalan IKRAM-UNITEN, 43000 Kajang, Selangor Malaysia; 7 UNITEN R&D Sdn. Bhd, Universiti Tenaga Nasional (UNITEN), Jalan Ikram-UNITEN, 43000 Kajang, Selangor Malaysia; 8 Department of Electrical, Electronic and Systems Engineering, Faculty of Engineering and Built Environment, Universiti Kebangsaan Malaysia, 43600 Bangi, Selangor, Malaysia; 9 Solar Energy Research Institute, Universiti Kebangsaan Malaysia, 43600 Bangi, Selangor Malaysia; 10 Department of Applied Mechanical Engineering, College of Applied Engineering, King Saud University, Muzahimiyah Branch, P.O. Box 800, 11421 Riyadh, Saudi Arabia; 11 Department of Mechanical Engineering and University Centre for Research & Development, Chandigarh University, Mohali, Punjab 140413 India; 12 Department of Mechanical Engineering, Faculty of Engineering, University of Malaya, 50603 Kuala Lumpur, Malaysia; 13 Department of Mechanical Engineering, Wolaita Sodo University, 4620 Wolaita Sodo, Ethiopia

**Keywords:** Energy, Decarbonization, Thin films, CdTe, Close-spaced sublimation, Chloride treatment, Energy science and technology, Materials science

## Abstract

An in-depth examination was undertaken on the structural, optical, and electrical alterations of CdTe thin films exposed to CuCl₂ post-treatment with different molar concentrations. The films were prepared using the close-spaced sublimation (CSS) approach. The CuCl₂ treatment at 390 °C in ambient air had significant impacts on the crystallographic orientation, lattice properties, and carrier concentration of CdTe films. The full width at half maximum (FWHM) increased from 0.0038 radians at 0.0005 M CuCl₂ to 0.0068 radians at 0.05 M, indicating grain refinement and increased microstrain at higher Cu concentrations, confirming a significant zincblende (111) preferred orientation as the X-ray diffraction (XRD) research reported. The inclusion of Cu and the resulting lattice deformation demonstrate a significant relationship with the evolution of crystallite size and dislocation density, indicating a regulated interaction between the dopant and the lattice throughout the examined molarity range. A comprehensive evaluation of structural, optical, morphological, and electrical properties indicates that a CuCl₂ concentration of 0.005 M achieves an optimal balance, facilitating effective p-type activation while preventing excessive Cu diffusion, which is associated with increased microstrain, decreased mobility, and heightened recombination at elevated dopant concentrations. XRD analysis confirms the retention of zincblende crystallinity without the formation of secondary phases, while optical measurements demonstrate stable band-gap behaviour, indicating minimal electronic distortion. Complementary FESEM and EDX analyses reveal enhanced grain coalescence and a reduction in grain-boundary density, which together facilitate improved carrier transport. The synergistic enhancements position the CuCl₂ solution-based treatment, especially at 0.005 M, as an effective and environmentally friendly alternative to traditional CdCl₂ activation, directly influencing the formation of ohmic contacts and the performance of CdTe thin-film.

## Introduction

As per the research findings by the International Renewable Energy Agency (IRENA) in conjunction with the Energy Agency for Photovoltaic Power Systems (IEA-PVPS), the growth of the cadmium-based photovoltaics industry may result in greater investment in waste management and recycling systems. Furthermore, crystalline silicon solar cell modules have a temperature coefficient of about − 0.50%/°C, whereas cadmium telluride thin films have a temperature coefficient of only − 0.25%/°C. This suggests that cadmium telluride devices are a better alternative in severe conditions such as low and high temperatures, and humid regions^1^. Based on the technological progress timeline, the recent research works on CdTe thin films mostly emphasize the comprehensive analysis and understanding of material properties to improve the carrier lifetimes, band grading, back contact formation, and doping-related limitations within the absorber region^2^. Good performance and stability of CdTe solar cells have been studied and discussed for a long period. However, a limiting factor in the quest for high-efficiency and durable CdTe devices is the open circuit voltage (V_OC_), which typically falls within the range of 800 mV. This limitation is attributed to the relatively low carrier concentration, self-compensation, and short lifetime in CdTe absorber region^3^. Consequently, it is suggested that the improvement of doping concentration at the CdTe polarity to be p-type can be beneficial. However, by increasing the doping concentration, the diffusion length can be reduced, which is detrimental to the charge carrier collection^4^. At the state of equilibrium, the product of the concentration of majority carriers and minority carriers remains constant, while the correlation among the concentrations of electrons, holes, and Fermi energy is elucidated by Eq. 1^5^.

1 $$\:{\mathrm{n}}_{0}{\mathrm{p}}_{0}={\mathrm{N}}_{\mathrm{C}}\mathrm{exp}\left(-\frac{{\mathrm{E}}_{\mathrm{C}}-\:{\mathrm{E}}_{\mathrm{F}}}{\mathrm{k}\mathrm{T}}\right)\:.\:{\mathrm{N}}_{\mathrm{V}}\mathrm{exp}\left(-\frac{{\mathrm{E}}_{\mathrm{F}}-\:{\mathrm{E}}_{\mathrm{V}}}{\mathrm{k}\mathrm{T}}\right)={\mathrm{n}}_{\mathrm{i}}^{2}$$

where *n*_*i*_ is the intrinsic carrier concentration and *n*_*0*_ and *p*_*0*_ are the electron and hole equilibrium carrier concentrations, respectively^6^. At the designated temperature, both *N*_*C*_ and *N*_*V*_ remain constant, *T* known as the effective density of energy states in the conduction and valence band. Meanwhile, *k* is the Boltzmann constant, *E*_*C*_ and *E*_*V*_ represent values of the conduction band and valence band accordingly, whereas *E*_*F*_ is known as the Fermi level. the intrinsic semiconductor, owing to its high purity devoid of impurities, maintains equilibrium wherein the quantities of charge carriers, electrons, and holes stand in equilibrium, denoted as *n = p*. Depending on the doping concentration, the relationship between the concentrations of the majority and minority carriers adheres to the principles outlined in the mass-action law^7^:

2 $$\:pn={N}_{C}{N}_{V}{exp}\left(-\frac{{E}_{g}}{kT}\right)={n}_{i}^{2}$$

where *E*_*g*_ is the energy gap between the conduction and valence band. When incident light interacts with a solar cell, photons possessing energy surpassing the band gap can induce the creation of electron-hole pairs within the solar cell. Ideally, the carriers dynamically transport through the drift-diffusion process mechanism^8^. Within the space charge region (SCR), the electric field strength and density accumulated as the net current flow is the sum of both diffusion and drift currents. Within the quasi-neutral region (QNR) situated in the absorber layer, the electric field exhibits negligible influence, with the net current flow predominantly governed by diffusion, dictating carrier mobility. Consequently, the diffusion length, representing the average distance a carrier traverses between generation and recombination events, is expressible through Eq. 3^9^.

3 $$\:{L}_{D}=\:\sqrt{D\tau\:}$$

where *D = µ(kT/q)* is the diffusion constant also known as Einstein relations, and τ the carrier lifetime both of which exhibit values pertaining to electrons and holes. Inadequate doping density restricts the maximum Voc that can be obtained from the device and very high doping concentration reduces the diffusion length but concurrently it increases the recombination rates^10^. In CdTe, the concentration of free charge carriers is predominantly regulated by intrinsic defects inside the crystal, such as vacancies and anti-site defects, which can counterbalance or neutralize introduced dopants, hence constraining the efficacy of external doping. Consequently, even with the introduction of external dopants, the carrier density may get influenced by defect chemistry and growth circumstances besides the dopant concentration^11^. During crystal growth the defective sites are partly incorporated into the solid phase, which means during solidification, the dopant concentration in the melt is not distributed evenly throughout the bulk and surface of the absorber layer^12^. According to Pfann’s theoretical studies, the fundamental aspect that needs concern is the effective segregation coefficient, which means during the crystal growth process, the impurity is incorporated into the melt. Therefore, the dopant concentration in the solid significantly increases during the crystallization process from the beginning to the end of the process^13^. In elemental semiconductors, the dopant atoms are often exclusively positioned at a substitutional or an interstitial site in the lattice. In some cases, a series of dopants in CdTe could occupy either normal atomic or interstitial sites. Therefore, the carrier in a crystal structure would collide with dopant atoms, lattice atoms, or a defect in the crystal structure^10^. As previously reported, as impurities transition from interstitial donors to substitutional acceptors, they initiate interactions with both extrinsic and intrinsic defects. This transition often triggers the creation of compensating intrinsic interstitial donor defects, consequently influencing the formation energy of point defects and, in turn, impacting device performance^14^. Copper (Cu) as a dopant can form an interstitial Cu_i_ and a substitutional Cu_Cd_ as donor dopant and acceptor dopant respectively^15^. Dopants, on the other hand, diffuse in CdTe crystals throughout the development process from the vapor, liquid, or solid phase, as denoted by the “effective diffusion coefficient” (D_eff_). In addition, D_eff_ highly corresponds to temperature as the diffusion mechanism occurs when significant activation energy is enabled^16^. Accordingly, the ultimate device performance can be significantly improved by optimizing both the dopant molarity and the applied heat during the process. CdCl_2_ treatment is a common method formerly used to improve CdTe thin films characteristics. However, the toxicity of Cd gas during the annealing process at high temperatures (400 $^{ \circ } {\mathrm{C}}$) induced the threat of EHS^17^. Cadmium telluride (CdTe) thin-film photovoltaics also often provoke concerns due to cadmium’s classification as a dangerous heavy metal; nevertheless, it is essential to differentiate between the chemical form of cadmium in CdTe and that in highly soluble cadmium salts, such as cadmium chloride (CdCl₂). CdCl₂ is one of the most toxic forms of cadmium due to its high-water solubility, propensity to dissociate and release Cd²⁺ ions, and significant bioavailability via inhalation or ingestion, resulting in well-documented acute and chronic toxicological effects on the lungs, kidneys, and skeletal system. Conversely, cadmium in CdTe is present within a robust crystalline semiconductor lattice distinguished by strong Cd–Te bonding, minimal solubility in water, negligible vapor pressure under ambient conditions, and high thermal stability, with decomposition or substantial cadmium release necessitating temperatures significantly exceeding those typical during standard usage. Issues with device sustainability, regulatory demands, and public perception have prompted significant research on cadmium-free thin-film photovoltaic materials that may entirely supplant CdTe while preserving high efficiency and scalability. Among second-generation thin-film technologies, copper indium gallium selenide (CIGS) solar cells have emerged as a prominent alternative, with efficiencies that are equivalent to or surpass those of cadmium telluride (CdTe), especially when combined with cadmium-free buffer layers such zinc oxide sulphide (Zn(O, S)) instead of cadmium sulphide (CdS). Kesterite-based absorbers, including Cu₂ZnSnS₄ and Cu₂ZnSnSe₄ (CZTS/CZTSSe), have garnered significant attention owing to their composition of earth-abundant and comparatively non-toxic elements; nonetheless, more study is necessary to address efficiency-limiting flaws and interface losses. In addition to these established thin-film systems, novel absorber technologies—such as lead-free and low-toxicity perovskite solar cells, organic photovoltaics, dye-sensitized solar cells, and cadmium-free quantum dot absorbers—are being rigorously investigated as means to completely eradicate cadmium from thin-film solar modules. Given these reservations, ongoing research on CdTe photovoltaics is scientifically warranted, as CdTe represents a commercially mature, market-validated thin-film technology characterized by long-term field reliability, a well-established large-scale manufacturing framework, and thoroughly documented life-cycle management and recycling processes, thereby serving as a pivotal benchmark for the progression of thin-film photovoltaic science. Furthermore, CdTe presently offers one of the lowest levelized costs of electricity and the highest industrial module efficiencies within thin-film technologies. Research on CdTe facilitates incremental enhancements in safety, efficiency, and sustainability that can be directly applied to both current CdTe production lines and developing Cd-free absorber systems. Regarding the doping alternatives, over the recent years, lots of doping strategies have been explored subjected to CdTe compounds using various materials and growth techniques to identify appropriate doping materials that might be the best fit for CdTe thin films characteristics^18–20^. Recently, the incorporation of group V elements including Arsenic (As) and phosphorus (P) was demonstrated, However, the doping process was performed at moderate temperatures by the metal-organic chemical vapor deposition (MOCVD) technique^21^. Notably, the solution-based CuCl_2_ treatment in high temperatures ($\:\ge\:$150$^{ \circ } {\mathrm{C}}$) matches the CdTe key characteristics optimizing the Voc and device performance stability of the device^22^. An in-depth inspection, however, is necessary to estimate the correct molar content of Cu in the doping profile^23^. This study presents a controlled solution-based activation strategy using CuCl₂, differing from earlier reports that depend on vapor-phase or low-temperature doping techniques. This approach allows for precise adjustment of dopant concentration while preserving the structural integrity of the CdTe lattice. By linking Cu molarity to crystallographic changes, defect passivation, band-gap stability, and charge transport characteristics, the research offers a thorough understanding of how high-temperature CuCl₂ treatment influences both immediate electrical improvements and compositional stability of CdTe absorbers. This integrated assessment bridges a critical gap in existing literature, where Cu incorporation is often evaluated in isolation or without addressing the temperature impact on the results of CuCl_2_ treatment, thereby establishing the practical relevance of the proposed treatment for next-generation photovoltaic technologies.

## Experimental methodology

The p-type wet treatment method in CdTe absorber layer can enhance the acceptor concentration. Enhancing carrier collection and boosting the built-in potential within the CdTe absorber region necessitates identification of the optimal concentration of active dopant species in the treatment solution^24,25^. This optimization process involves a delicate balance, ensuring that the concentration is neither too low to provide adequate enhancement nor too high to risk detrimental effects on the device’s overall performance. In this work, the copper (Cu) concentration in CuCl_2_ solution was analyzed to control Cu incorporation in CdTe. Specifically, CdS/CdTe stacks featuring a CdS thickness of 100 nm and CdTe 3.0 μm were prepared on 3 × 3 × 0.1 cm ITO-coated borosilicate glass substrates using RF magnetron sputtering and close-spaced sublimation (CSS) process as shown in supporting Fig. 1(a) and (b), respectively. The CSS fabrication was conducted using high temperatures of 625 °C and 595 °C for source and substrate in 1.5 Torr Argon gas environment. The CdTe substrates were then activated by the CuCl_2_ wet solution. Before the process, CuCl_2_ is diluted in 25 mL of deionized (DI) water and divided into five sample groups. Five distinct solutions are formulated, each containing different molar concentrations of copper, ranging from 0.05 M to 0.0005 M. The CuCl₂ concentrations were selected on a logarithmic scale (0.0005 M to 0.05 M) rather than at uniform linear intervals. The rationale is that dopant incorporation and defect chemistry in compound semiconducting thin films typically exhibit non-linear responses with respect to precursor concentration, particularly near solubility limits or defect formation thresholds. A log-scale sampling approach allows the identification of both low-concentration regimes—where subtle improvements in conductivity and defect passivation may first emerge—and high-concentration regimes, where excessive dopant incorporation may induce scattering centers, secondary phase segregation, or structural disorder^23^.

**Fig. 1:**
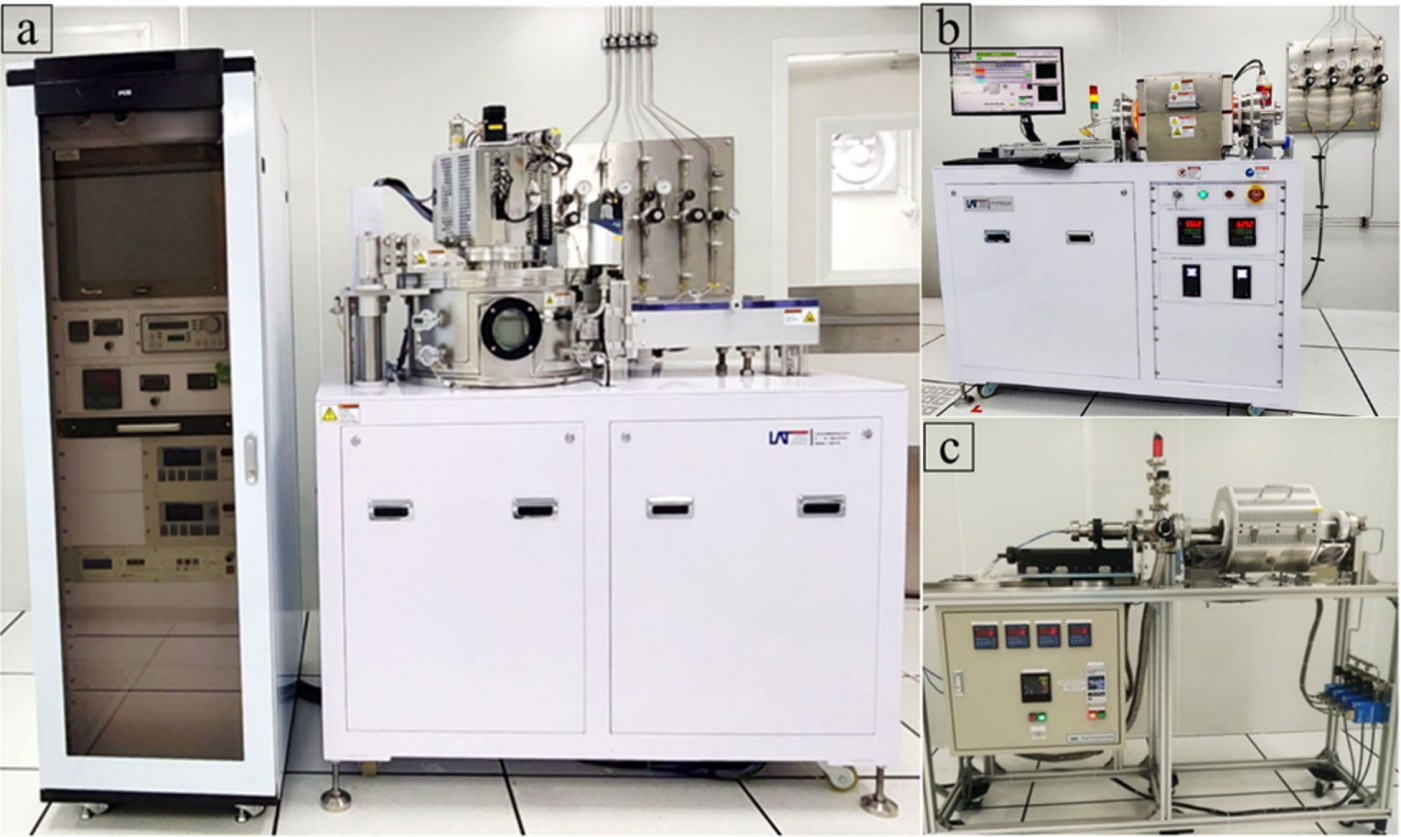
**a** Sputtering, **b** CSS and **c** annealing systems used in this study.

**Fig. 2:**
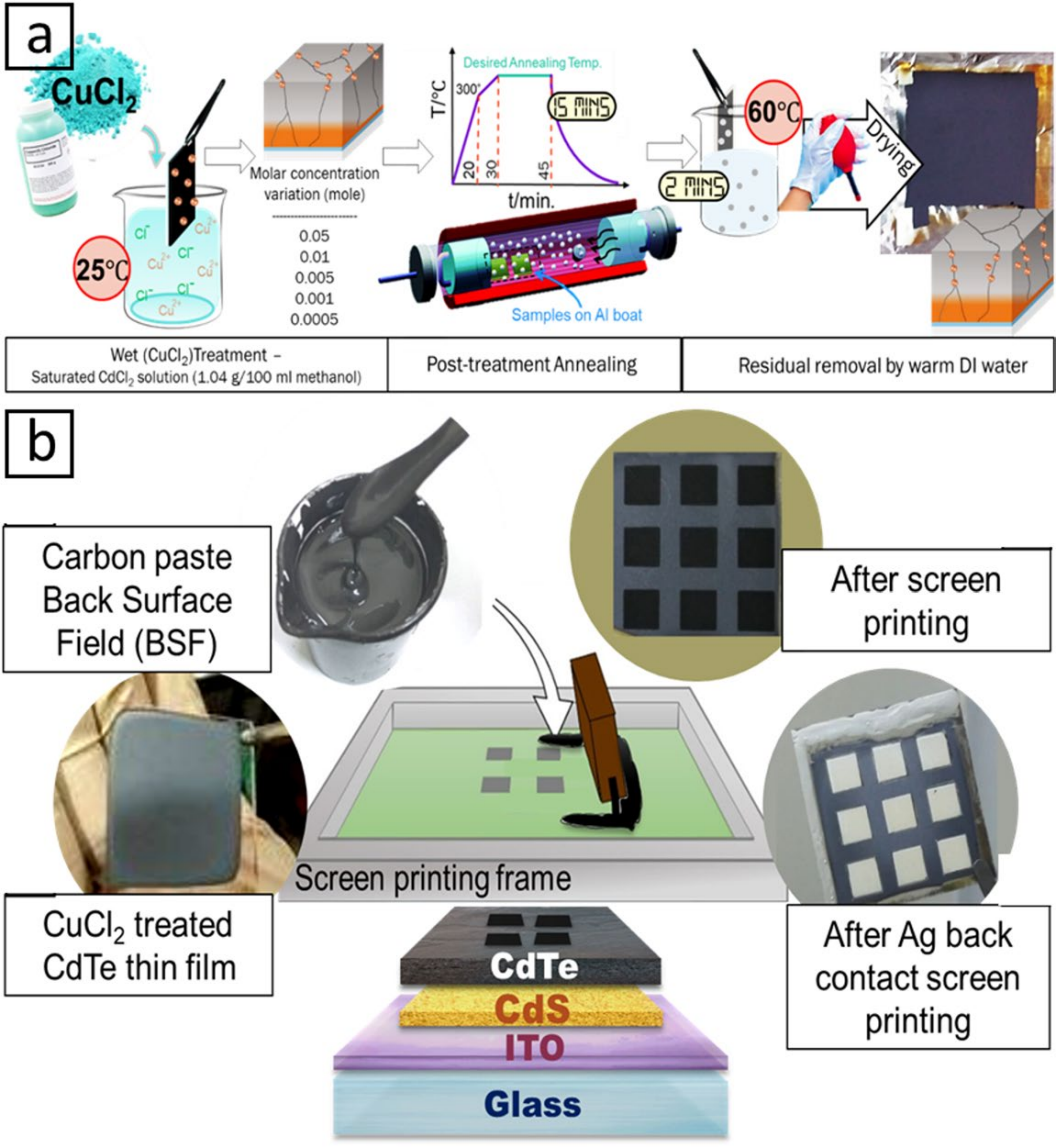
**a** CuCl_2_ post-treatment procedure on CSS-CdTe samples, **b** Complete CdTe thin films device and contact printing.

Subsequently, the CdS/CdTe substrates were immersed into the CuCl_2_ solutions and left to sit for 2 min at room temperature to allow tailored surface reaction with copper and chloride species. Afterward, the substrates were submerged in warm (60 °C) DI water to rinse the weakly bound species from the surface and dried the samples with a general N_2_ gas stream, followed by annealing at a temperature of 390 °C for 15 min in the open-air environment using the annealing system shown in Fig. 1(c) for each group of samples. The process for successful doping and annealing the samples using copper (II) chloride (CuCl₂) is demonstrated in Fig. 2a^26^. The decision to use 390 °C is driven by its demonstrated efficacy in facilitating chloride-assisted recrystallization, grain-boundary decoration, and defect passivation in CdTe absorber layers, processes necessitating transient thermal activation exceeding moderate annealing temperatures (e.g., 300–330 °C) for optimal effectiveness. At this temperature, chlorine species have enough mobility to adorn grain boundaries and promote localized stoichiometric reordering, whereas copper can function as an acceptor without provoking uncontrolled diffusion or secondary phase development, provided the dwell duration is suitably constrained.

CuCl₂ was dissolved in deionized water without pH adjustment and utilised immediately to prevent hydrolysis or complexation. Under these circumstances, Cu²⁺ stays largely in the hydrated ion form [Cu(H₂O)₆]²⁺ with only modest hydrolysis at neutral pH. This is typical for low-concentration CuCl₂ solutions made freshly. Furthermore, during coating and subsequent high-temperature annealing, the solvent evaporates and the Cu species are incorporated into the CdTe lattice or grain boundaries, where their chemical state is primarily determined by the host matrix and thermal treatment rather than the initial aqueous speciation.

In this study, the back contact was formed by screen printing of a conductive paste (the 1000 ppm ratio of copper-doped carbon paste (C: Cu)) followed by 60 min of heating in the oven to dry the paste as presented in Fig. 2b. Finally, the CdTe substrate samples underwent analysis to assess their structural, optical, and electrical characteristics.

Crystalline properties and structure as well as crystal orientation along the CdTe thin films surface were examined by Shimadzu XRD-6000 Advance Cu-Kα diffractometer at room temperature. X-ray diffraction (XRD) patterns were captured within the 2θ range of 20º to 80º, employing Cu-Kα radiation wavelength, with a precise step size of 0.02º, λ = 1.5408 Å, voltage, V = 40 kV and current, I = 40 mA. Grain size, surface morphology and cross-sectional view and thickness were observed by using Hitachi SU8030 field emission scanning electron microscope (FESEM) which was operated at 3 kV to 15 kV. The obtained images underwent thorough processing and analysis utilizing both 2D and 3D ImageJ software (version 1.54d), created by Wayne Rasband and colleagues at the National Institutes of Health (NIH), USA. The app operated on a 64-bit machine utilizing Java 1.8.0_345. ImageJ is a publicly available, open-source framework for image analysis, accessible at https://imagej.org. This comprehensive approach allowed for a meticulous evaluation of the surface quality, enabling a detailed examination of sample characteristics and features in both two and three dimensions. The compositions of the CdTe thin films were obtained by energy dispersive spectroscopy (EDX) using a multi-dimensional Helios system with operating parameters of 15 keV, and the average working distance between the sample and the signal lens was 3–15 mm. Optical transmission and absorption measurements were performed at room temperature by utilizing Perkin Elmer Lambda 35 UV-Vis spectrophotometer starting at the wavelength of 200 to 1000 nm. The optical transmission and absorption measurement were applied from 300 to 1000 nm wavelength range through CdTe layer and CdS layer. Energy band gap values were calculated from the obtained transmission and absorption spectra. The electrical characteristics, including charge carrier concentration, resistivity, and mobility were tested by HMS Ecopia 3000 Hall-Effect measurement system with 0.57 T of magnetic field and probe current in the range of 40 nA to 10 mA for all prepared samples. As a result, measurements were conducted following standard characterization protocols with calibrated instruments to ensure data reliability.

## Results and discussion

**Fig. 3:**
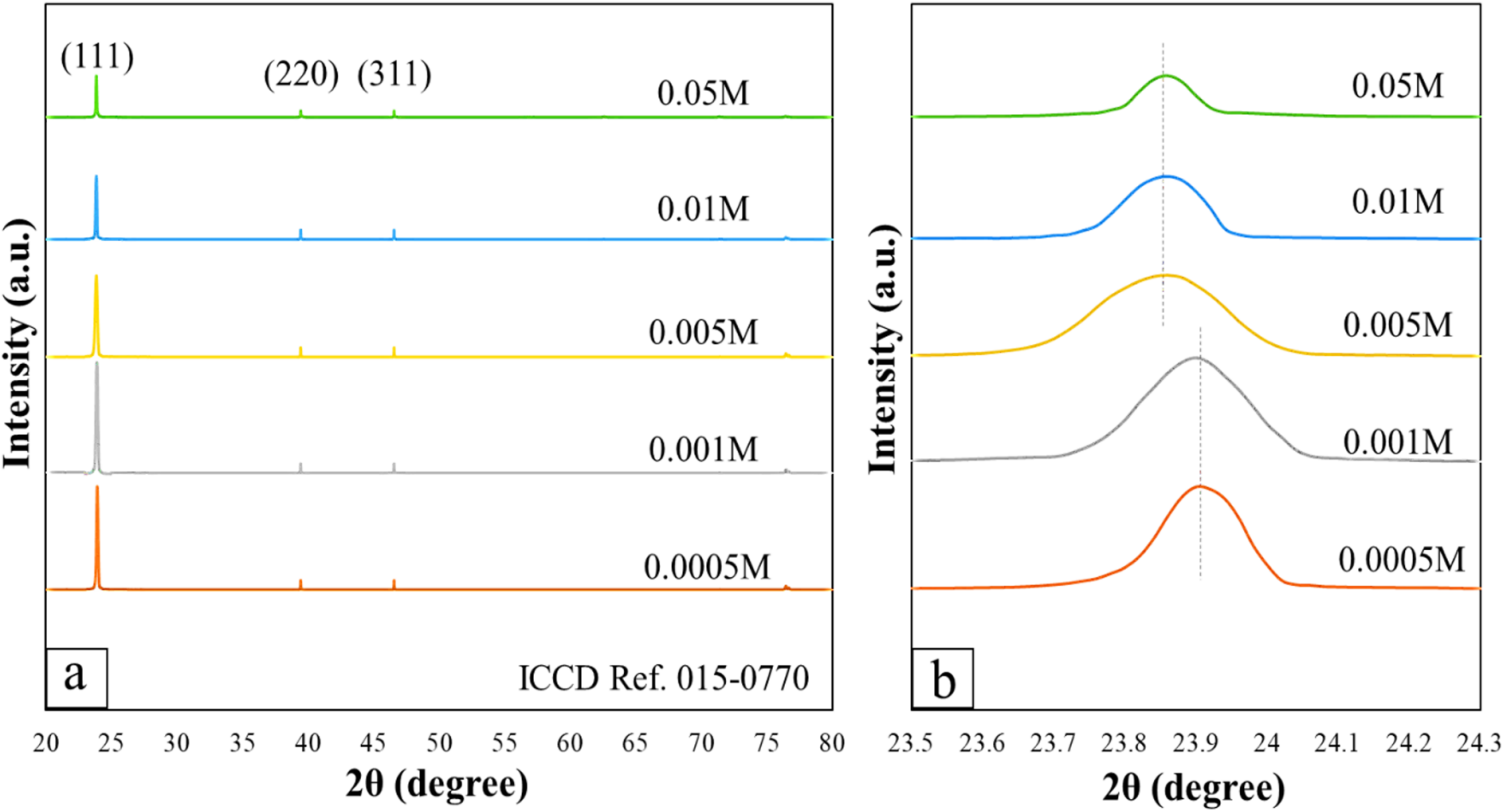
**a** XRD spectra of CdTe thin films with CuCl_2_ from 0.0005–0.05 M, **b** main peak focused view.

The comparative crystallite structural properties of CdTe thin films by CuCl_2_ wet treatment method is examined by XRD pattern spectra analysis recorded from 20º to 80º as shown in Fig. 3. XRD analysis shows that for all samples, only the cubic CdTe peaks with (111) preferential orientation are observed. Peaks at 23.75º, 39.29º, and 46.51º correspond to (111), (220), and (311) planes of the polycrystalline cubic zinc blende structure CdTe respectively. The CdTe thin films peak orientation is in good agreement with ICCD Ref. 015–0770. As predicted for a dilute dopant, any change in the average lattice parameter is negligible, therefore peaks in the overview graph appear nearly identical. Magnifying and fitting the (111) reflection reveal tiny but consistent variations over the CuCl₂ series. The low-concentration sample (0.001 − 0.0005 M) has a somewhat greater 2θ than the high-concentration sample (0.05–0.05 M), with the latter shifting to a lower angle. The absolute magnitude of these shifts is small, indicating dilute doping. The procedure adds dopant-level amounts of Cu (and Cl) to CdTe rather than modifying its composition, as in an alloy. According to Bragg’s low, a shift of Δ(2θ) ≈ 0.02–0.06° at 2θ = 23.7° corresponds to |Δa|/a of ~ 0.05–0.2%, indicating a minor change in the lattice constant. Such magnitudes are prevalent when the dopant concentration is maintained below the threshold necessary for significant alloying or the emergence of a new phase. These changes are visually minor in an overview plot Fig. 3a, but are clearly resolved in the enlarged view Fig. 3b.

Employing optimal (390 $^{ \circ } {\mathrm{C}}$) activation temperature during the 15-minute annealing process after submerging the samples in the CuCl_2_ solution^22,27^, the diffusion process is enhanced and presented in XRD results. The 0.0005 M and 0.001 M samples exhibited higher intensity of (111) preferential orientation with peaks other than (111) barely apparent and lower peak intensity of (111) orientation corresponds to 0.05 M and 0.01 M samples. Previous reports confirmed that the energy band barrier could be reduced and the acceptor concentration within the absorber layer increases by the CuCl_2_ annealing process^28^. Figure 3 (b) depicts the onset of structural alterations in treated samples, occurring once the copper concentration surpasses 0.005 M. This peak shift indicates dopant-induced lattice distortion rather than the formation of new phase^29^. No secondary phases related to Cu-Te or Cl-containing compounds were detected. This demonstrates that the CuCl₂ treatment operates as doping rather than alloying or creating a changed phase. It could indicate the uniform distribution of Cu in polycrystalline CdTe structure with a lattice constant of 6.477 nm similarly reported in^30,31^. At lower CuCl₂ concentrations, substitutional Cu on Cd sites causes a slight lattice contraction, shifting the peak to higher 2θ angles. Still, the variations in lattice parameters are within acceptable limits, with no changes in the composition of the host substance, as Cu is incorporated at dopant-level concentrations, thereby preserving the fundamental zincblende CdTe crystal framework. Alternatively, excessive copper incorporation leading to alloying would likely to cause substantial and systematic changes in lattice parameters due to significant compositional changes. This could further be accompanied by notable peak shifts, increased peak asymmetry, and/or the formation of secondary phases related to Cu–Te compounds. Such excessive amount of copper doping at the alloying level could potentially lead to more significant lattice distortion, heightened strain accumulation, and a marked deviation of the lattice constant from that of pure CdTe. The absence of these features in the current XRD results, coupled with the trends in controlled peak broadening (FWHM), suggests that the structural changes observed stem from microstrain induced by the dopant and the refinement of crystallite size, rather than alloy formation.

By implementing the Scherrer equation as well as full-width half maximum (FWHM) analysis through the preferred orientation (111) from the XRD spectra plot, the crystallite structural properties such as crystallite size could be determined by Scherrer’s formula as presented in Table 1^32^.

**Table 1:** Calculated structural aspects of CdTe samples with molar CuCl_2_ variations.

Molarity	Plane (hkl)	FWHM, β (radian)	Crystallite size, D (nm)	Lattice constant a_cubic_ (nm)	Microstrain, ε (×10^− 3^)	Dislocation density, δ (×10^11^ cm^− 2^)
0.05 M	(111)	0.0068	21.382	0.6520	7.813	2.187
0.01 M	(111)	0.0066	21.377	0.6524	7.854	2.188
0.005 M	(111)	0.0059	23.895	0.6510	7.009	1.751
0.001 M	(111)	0.0042	33.855	0.6490	4.933	0.872
0.0005 M	(111)	0.0038	36.934	0.6488	4.520	0.733

Subsequently, using the crystallite size values, the micro-defect density at crystallite scale for (111) orientation can be calculated as well. As demonstrated, decreasing crystallite size increases the dislocation density and lattice misfit. Here the lattice constant a_cubic_ is also affected slightly by the dopant density and its value is calculated by Eq. 4^33^.

4 $$~{\mathrm{a}}_{{{\mathrm{cubic}}}} = {\text{ d}}_{{{\mathrm{hkl}}}} \left( {{\mathrm{h}}^{{\mathrm{2}}} + {\text{ k}}^{{\mathrm{2}}} + {\text{ l}}^{{\mathrm{2}}} } \right)^{{{\mathrm{1}}/{\mathrm{2}}}}$$

Here the *h*,* k*, and *l* are the Miller indices and *d*_*hkl*_ denotes the inter-planar spacing of a neighboring crystal plane. With increasing dopant molarity, the lattice constant of treated samples nears the lattice constant of perfect cubic zincblende-oriented CdTe thin films, which is reported to be 6.482 nm. This is due to the suppression of defect points in the bulk of CSS-grown CdTe toward increased crystallinity^34^. Table 1 also highlights the highest crystallite grain size of (111) preferential orientation is 36.934 nm at 0.0005 M while the lowest 21.382 nm is 0.05 M accordingly. There is a noticeable change of crystallite grain size for different CuCl_2_ molar concentrations, specifically for treated 0.001 M and 0.0005 M samples where the major crystallite size increase is observed. This increase took place in favor of the increase in favorable cubic crystal orientation resulting from the XRD test^27^. Overall, the most cubic-oriented samples resulting from XRD graph were found to be the treated samples by 0.001 M and 0.0005 M solutions.

**Fig. 4:**
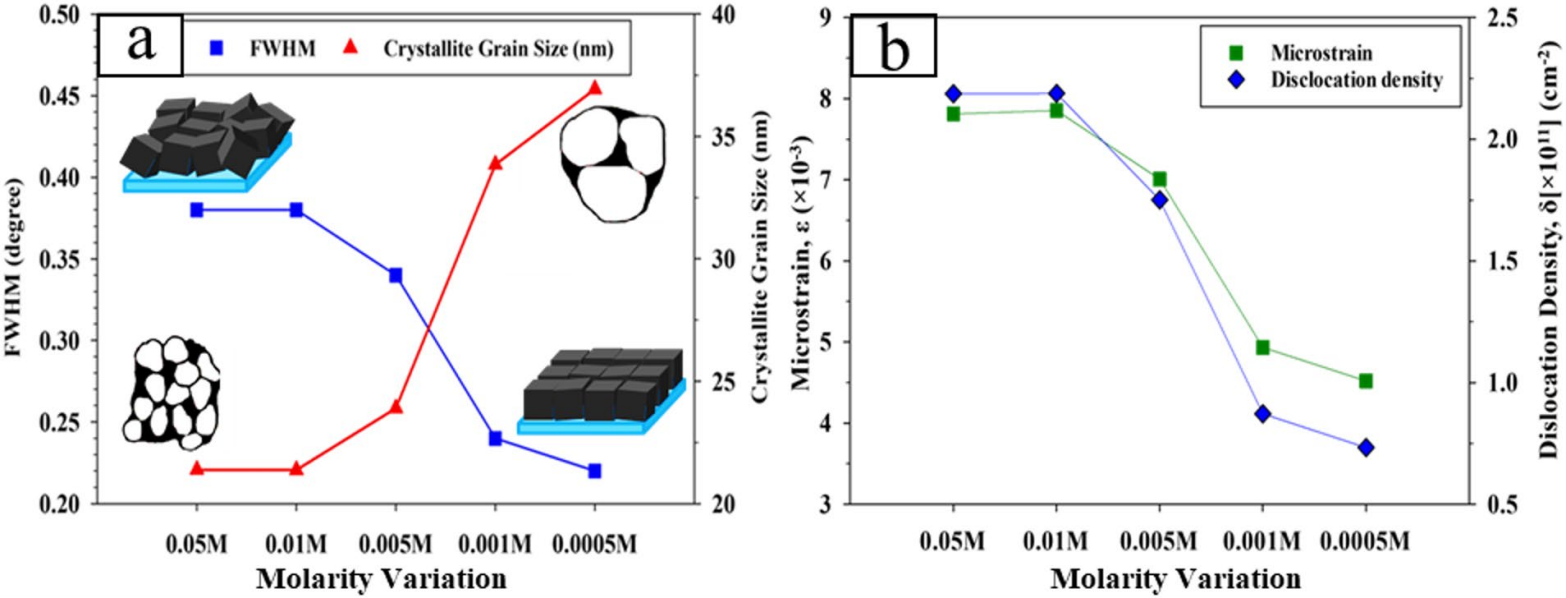
**a** FWHM and crystallite grain size and **b** Micro-defect density, for CdTe samples treated by different CuCl_2_ molar concentrations.

Figure 4(a) displays a correlation between FWHM and crystallite size at (111) orientation of CuCl_2_-treated CdTe thin film. The crystallite grain size ranged from 21 to 37 nm. Notably, the lower concentration of copper in the treatment solution yielded a highly cubic crystal orientation, aligning with expectations. This outcome can be attributed to factors including reduced surface energy, minimal impact on nucleation and growth rates, fewer nuclei formation, and a lower presence of additives or impurities within the host material (CdTe)^35^. Figure 4(b) displays the comparison of microstrain and dislocation density of the (111) peak orientation of CdTe thin film for CuCl_2_ wet treatment. It shows the microstrain of the crystal structure declines similarly with dislocation density as the molar concentration becomes low. The highest microstrain (7.854 × 10^− 3^) and maximum value of dislocation density (2.188 × 10^11^ cm^− 2^) was obtained for CuCl_2_ molar concentration 0.01 M. Microstrain and dislocation density increased mainly in samples treated with highly concentrated CuCl_2_, as predicted due to the introduction of a significant number of impurities or dopants, resulting in a change in the lattice structure^36^. However, the shift in the lattice is observed to be toward the highly crystallite CdTe which is favorable for enhanced optical absorption in the prepared samples^37^. Ultimately, the structural analysis revealed an efficient doping process in which the grains are increasingly influenced by the increase in dopant molarity. Several first-principles investigations have offered a detailed theoretical explanation of how increasing CdCl₂ or CuCl₂ dopant molarity increases the crystallinity of CdTe, which supports the experimental data in this work. Chatratin, performed hybrid-DFT (HSE06) simulations in VASP to show that Cl drives self-compensation in bulk CdTe while efficiently passivating deep defect centres, which explains the observed crystallinity increase at optimum dopant levels^38^. Similarly, Park et al. integrated SIESTA (PBE) and VASP (HSE06) frameworks to indicate that Σ3 grain boundaries become structurally and electrically benign with Cl inclusion^39^. This coincides with the decrease of defect spots seen experimentally.Another report extended this insight by using hybrid-DFT (HSE06) in the VASP (MedeA) environment to uncover low-energy Cu-Cl-native-defect compounds that neutralise deep traps, explaining the better ordering seen with modest dopant incorporation^40^. Sharmin et al. also employed Quantum ESPRESSO and WEST to demonstrate that chloride activation efficiently passivates Te antisites and promotes lattice relaxation, lowering microstrain and peak widening in XRD patterns^41^. Sundar et al. confirmed that Cl preferentially accumulates at grain borders, minimising harmful gap states and promoting local structural order^42,43^. Furthermore, as previously reported in DFT research works^44^, optimum doping in CdTe contacts alters electronic structure and defect states, hence improving interfacial characteristics. The current study confirms that appropriate CdCl₂ doping enhances CdTe crystallinity, reduces defect density, and affects lattice strain, aligning with simulation expectations.

**Fig. 5:**
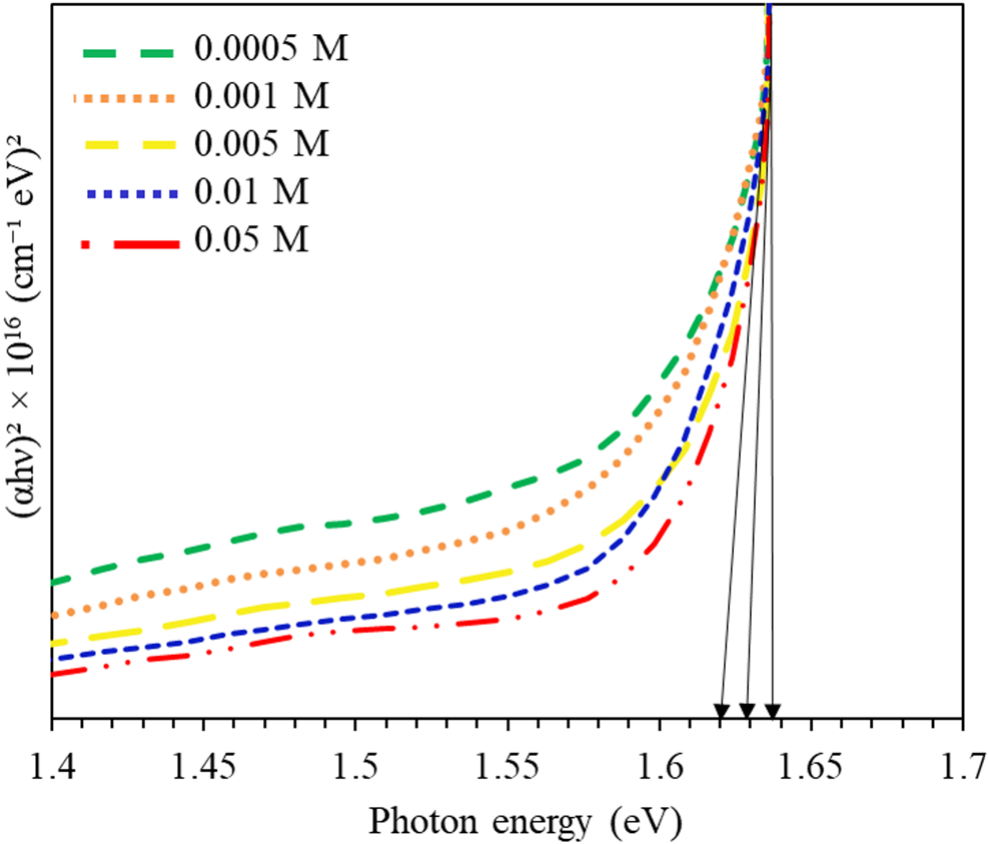
Tauc plot of CdTe thin films for CuCl_2_ molar variations.

In addition, to measure the absorption index of the deposited thin films, optical properties of CdTe thin films treated by different molar concentrations of CuCl_2_, UV-Vis spectroscopy was utilized. By applying the absorption index values, the energy band (Eg) of treated samples was calculated as demonstrated in Fig. 5.

The band gap is calculated by the relation αhν = Aº(hν- Eg)^n^ and Tauc plot which can be displayed by tangent lines through the plot^22^. Samples treated with varying CuCl_2_ molar concentration depositions exhibited the band gap values in the range of 1.62 to 1.64 as shown in Fig. 5. The observed rise in the optical band gap from 1.62 eV to 1.64 eV with increasing CuCl₂ concentration, despite predicted lattice expansion, can be attributed to a mix of causes. First, the introduction of Cu into the CdTe lattice results in Cu-induced defect states, which can form localised energy levels inside the band gap, modifying the electrical structure and expanding the band gap. Furthermore, Cu near the grain boundaries may affect the material’s electrical characteristics, adding to the observed shift. Finally, Cu d-states, which are known to interact with CdTe’s conduction band, are likely involved in changing the material’s electrical structure, adding to the rise in the optical band gap.

**Table 2:** Band gap energy of CdTe thin films for CuCl_2_ molar variations.

Molarity	Band gap (E_g_)
0.05 M	1.64 eV
0.01 M	1.63 eV
0.005 M	1.62 eV
0.001 M	1.62 eV
0.0005 M	1.62 eV

Table 2 shows that the optical band gap of CdTe films widens somewhat with increasing CuCl₂ concentration, from 1.62 eV at low concentrations (0.0005–0.005 M) to 1.64 eV at the maximum concentration of 0.05 M. Such alterations, albeit small in absolute value, are compatible with dopant-induced changes to the semiconductor’s electrical structure. At greater Cu loadings, localised quantum confinement effects become more evident as the dopant changes grain size and electronic potential landscapes, moving the absorption edge to higher energies. Furthermore, Cu insertion creates point defects that can disrupt the local crystallographic arrangement, contributing to further band gap widening via defect-related electronic states^45^. However, when dopant density increases, interactions between nearby dopant atoms become more dominant, altering the electronic band structure and partially compensating for the initial widening. This mix of quantum confinement, defect-induced effects, and dopant-dopant interactions accounts for both the apparent rise in band gap at greater concentrations and the propensity to saturation or narrowing beyond the optimum doping range. Several works are previously reported on Cu-doped CdTe thin films consistently indicate that the optical transitions retain their direct character regardless of variations in Cu concentration^18–21^. These discoveries align with the intrinsic electrical structure of CdTe, acknowledged as a direct band gap semiconductor in both undoped and doped states, rendering it appropriate for solar applications.

**Fig. 6:**
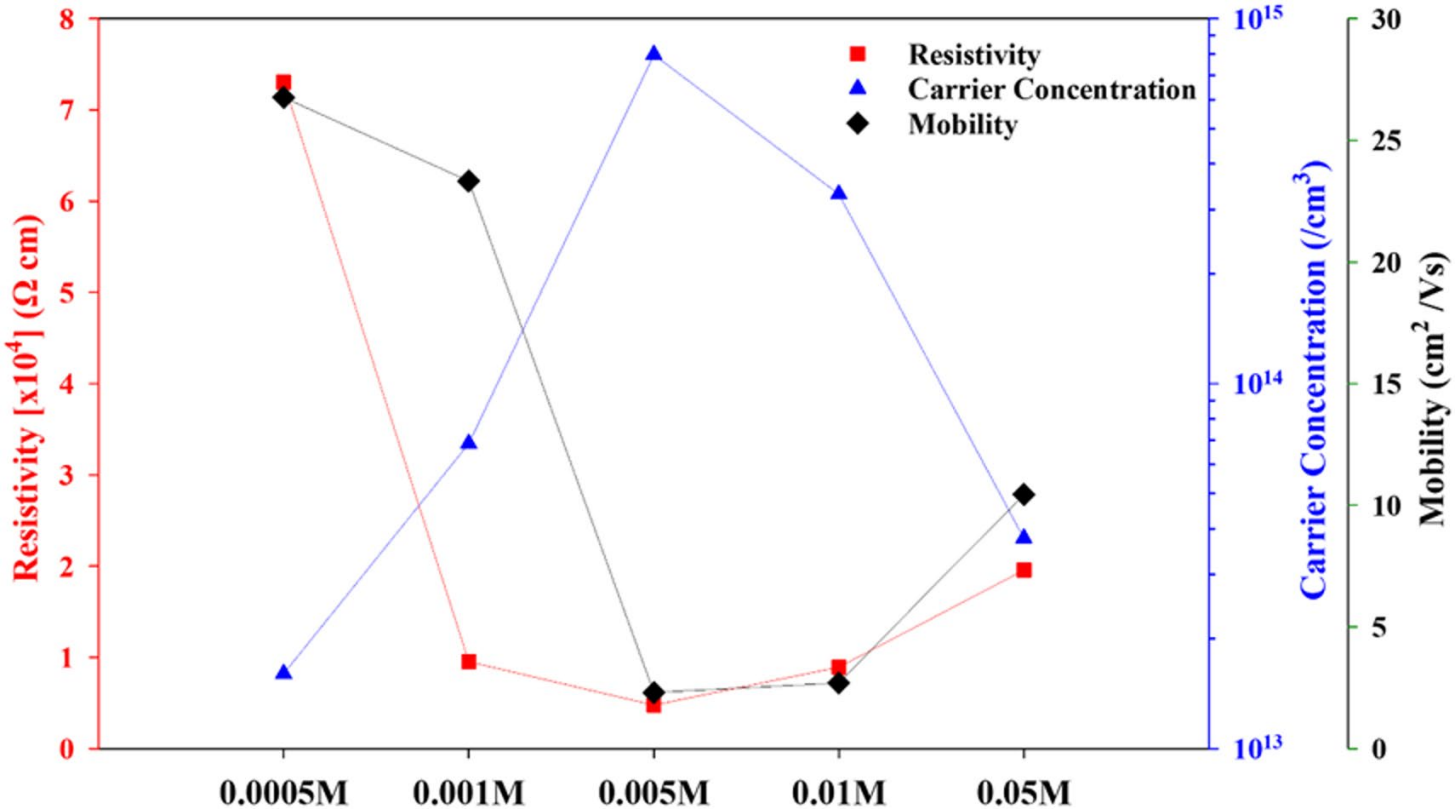
Electrical measures of CdTe films after CuCl_2_ molar variations.

For electrical attributes, a Hall-Effect measurement system is employed on the CdTe layers at room temperature environment. The 0.57 T magnitude of the magnetic field and 40 nA of the current source are applied in this evaluation. Consequently, this investigation enables the deduction of carrier concentration, mobility, and resistivity. All thin films demonstrate carrier concentration in a range of 10^13^− 10^14^ cm^− 3^ accordingly. CdTe thin films sample doped with 0.005 M CuCl_2_ exhibit higher carrier concentration compared to other samples presented in Fig. 6; Table 3 respectively. The variation in Hall mobility, which decreases at low CuCl₂ concentrations and increases at higher concentrations, may be explained by the interaction of carrier scattering and carrier concentration. At low Cu concentrations (0.0005 M to 0.01 M), Cu-induced defects and grain boundary scattering dominate, resulting in enhanced carrier scattering and reduced mobility. However, as the Cu concentration rises (0.05 M or higher), carrier concentration and activation improve due to more effective doping and lower scattering, resulting in increased mobility. This behavior is consistent with prior work on doping in II-VI semiconductors, where low dopant concentrations can enhance defect scattering, while higher concentrations can lead to superior electrical characteristics through more effective carrier activation and fewer scattering centers.

As the CdTe compound undergoes doping, the carrier mobility experiences a notable decrease due to the scattering centers for charge carriers, leading to increased collisions and ionized impurities that may act as charged scattering centers within the crystal lattice^46^. Moreover, the fabrication of functional CdTe devices can be greatly affected by the positioning of donors or acceptors within the crystalline lattice, giving rise to various energy states associated with the CdTe lattice. This intricate interplay of dopants and their arrangement influences the electrical potential of the material. Notably, the observed lowest carrier concentration of 2.6 × 10^13^ cm^− 3^ in a sample treated with 0.0005 M CuCl_2_ signifies an inadequacy of copper content to effectively generate carriers in the CdTe samples. This deficiency in the dopant concentration may hinder the desired electrical characteristics needed for optimal device performance. Achieving the right balance in doping levels is pivotal for ensuring efficient carrier generation and subsequently enhancing the overall performance of CdTe solar cells^40^. overall, the carrier concentration falls within a comparable range, approximately 10^14^ cm^− 3^ as typical for Cu-doped polycrystalline CdTe thin films^46^. The results from the baseline sample before the treatment presented in Table 3 clarify the significance of the treatment process^44^.

**Table 3:** Electrical properties of CdTe absorbers with CuCl_2_ molar variations.

Molarity	Carrier concentration (/cm^3^)	Carrier concentration (After 1 year) (/cm^3^)	Mobility (cm^2^/Vs)	Resistivity [x10^4^] (Ω cm)
Baseline	1.10 × 10^13^	1.00 × 10^12^	5.5	10.8
0.05 M	3.77 × 10^13^	3.00 × 10^13^	10.44	1.96
0.01 M	3.31 × 10^14^	3.11 × 10^14^	2.697	0.89
0.005 M	7.97 × 10^15^	5.30 × 10^15^	2.302	0.48
0.001 M	6.85 × 10^13^	3.48 × 10^11^	23.33	0.95
0.0005 M	2.60 × 10^13^	1.00 × 10^11^	26.76	7.30

**Fig. 7:**
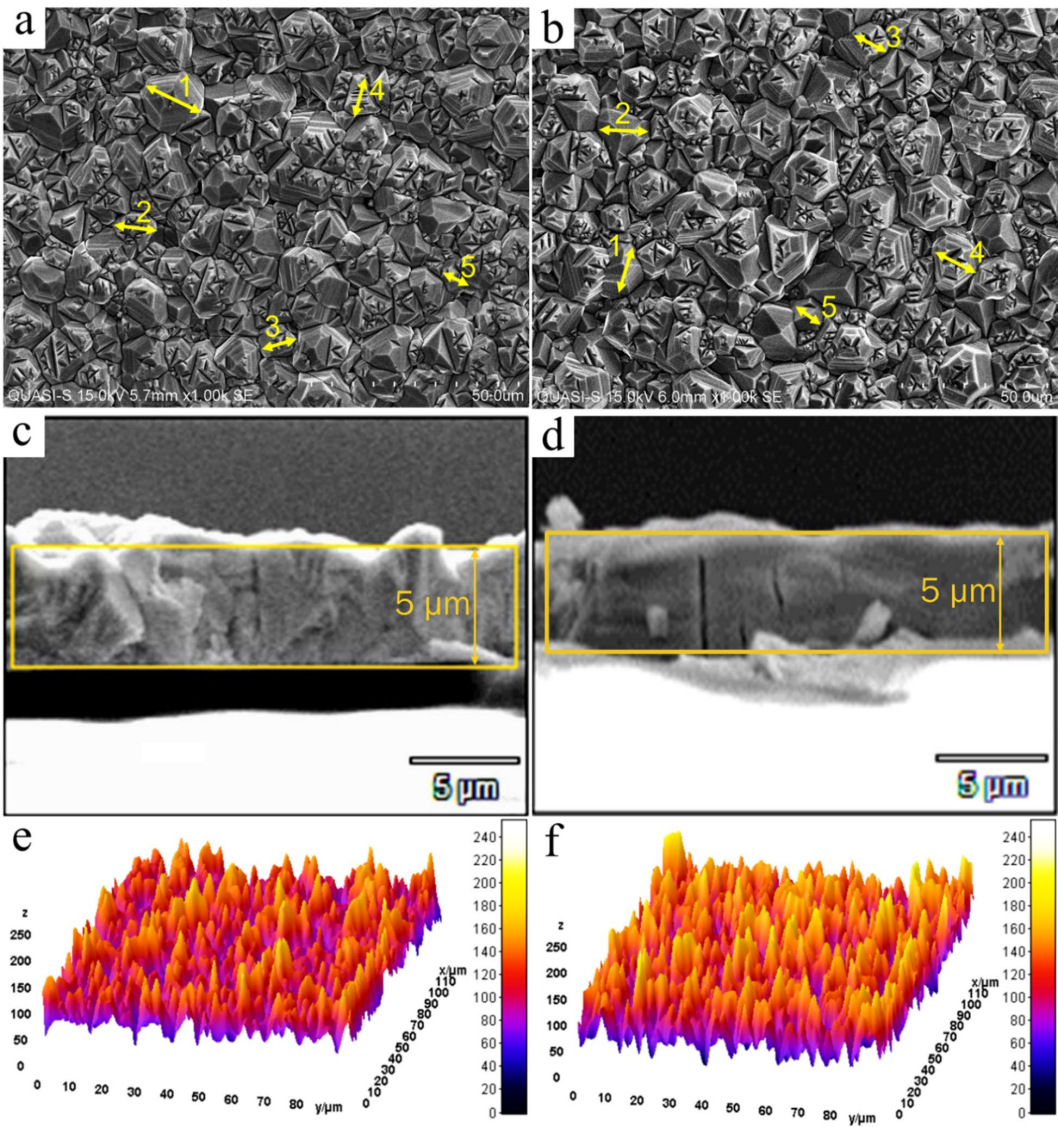
FESEM results including the top view of **a** baseline, **b** treated CdTe, cross-sectional view of **c** baseline **d** treated CdTe, and the 3-Dimensional morphology of **e** baseline and, **f** treated CdTe.

The resistivity value doesn’t exceed 2.0 × 10^4^ Ωcm except for the samples with molar concentration 0.0005 M CuCl_2_ which increases to 7.3 × 10^4^ Ω-cm. The sample cultivated in a 0.0005 M diluted CuCl_2_ solution displays the most remarkable mobility which also exhibits the highest resistivity. Cu dopants are not engaged as an active acceptor in the 0.001 M and 0.0005 M samples, and as illustrated by XRD measurements the films with lower Cu concentration (more or less than the adequate density) appear to have better crystal orientation near to the optimal CdTe thin films however the Cu primarily was bonded with Te atoms which resulted in a highest XRD peaks and lower electrical properties, as reported in the literature^47^. Overall, Cu inclusion and subsequent annealing in doped semiconductor thin films can affect both microstructure (grain growth, defect passivation, scattering centres) and electronic states (dopant activation). Mobility and carrier concentration frequently fluctuate in opposing directions: higher scattering reduces mobility, whereas more active dopant sites increase carrier concentration. This equilibrium often results in resistivity being almost constant. Following this test, to monitor the effect of excessive doping and Cu interdiffusion, all samples to be tracked were preserved in a desiccator for 360 days until the day of analysis, and then the carrier concentration measurement was performed in room temperature using the same Hall measurement system as shown in Table 3. It is also worth mentioning that the gradual carrier density reduction by the increment of copper dopant molarity derived from samples doped with 0.01 M and 0.005 M was caused by excessive Cu diffusion, which shall be investigated further. Furthermore, the morphological changes in CdTe samples were analysed using FESEM and EDX both Baseline CdTe samples before undergoing CuCl_2_ treatment, and the optimally doped samples with 0.005 M Cu. This analysis aimed to illustrate the alterations in the sample’s elemental and morphological characteristics following an effective treatment process. The cost of cadmium telluride device production is very low, and the current component cost can be around $ 0.40/W^48^. Therefore, in emerging technologies, the cadmium telluride device share continues to increase. Recombination centres within the CdTe absorber itself, i.e., defects at the grain boundaries or even in-grain defects, can seriously alter the performance of the devices. Thus, as the conclusive measure to examine alterations in composition and morphology leading to the enhanced optoelectronic properties of CuCl_2_-treated CdTe in contrast to untreated samples, Fig. 7 displays the FESEM findings. The top view of the before and after treatment samples in Fig. 7(a) and (b) evidence a notable grain growth and cross-sectional images 8(c) and 8(d) illustrate anomalous grains and the growth mechanism that rectifies these anomalies seamlessly, resulting in a dense, contiguous structure, pertinent to material science aficionados. After treatment, respectively. The grain boundaries are passivated with chlorine under the meticulously controlled diffusion of CuCl_2_ along the grain boundaries during the activation treatment, improving the collection efficiency of the photogenerated carriers resulting as presented in Fig. 7, the Hall effect results. Figure 7(d) demonstrates the creation of continuous, thin, and homogeneous films, a phenomenon influenced by various factors, including nucleation and growth mechanisms, as well as the amalgamation of diminutive crystallites facilitates the mitigation of structural tensions and surface energy, thereby fostering a prevailing propensity for orientation predominantly along the (111) plane, in line with the previous reports^49,50^. Also, the formation of larger grains is observed to result in a decreased strain value in treated samples as corroborated by the XRD results presented in this study. This is ascribed to the minimized grain boundaries and defects in treated samples, illustrated in Fig. 7(e) and (f).

Table 4 reveals that the average grain area size is 0.956 μm² before treatment, and this increases to 1.116 μm². This indicates a reduction in the grain boundaries, which are the points of recombination.

**Table 4:** Average grain size in the area and length measures.

Sample Points	Base CdTe		Optimal CuCl_2_ Treated CdTe	
	Area (µm^2^)	Length (µm)	Area (µm^2^)	Length (µm)
1	1.466	14.688	1.309	13.143
2	1.063	10.615	1.358	13.580
3	0.856	8.490	0.945	9.409
4	0.787	7.814	1.191	11.855
5	0.610	6.039	0.777	7.700
Average	**0.956**	**9.529**	**1.116**	**11.137**

**Fig. 8:**
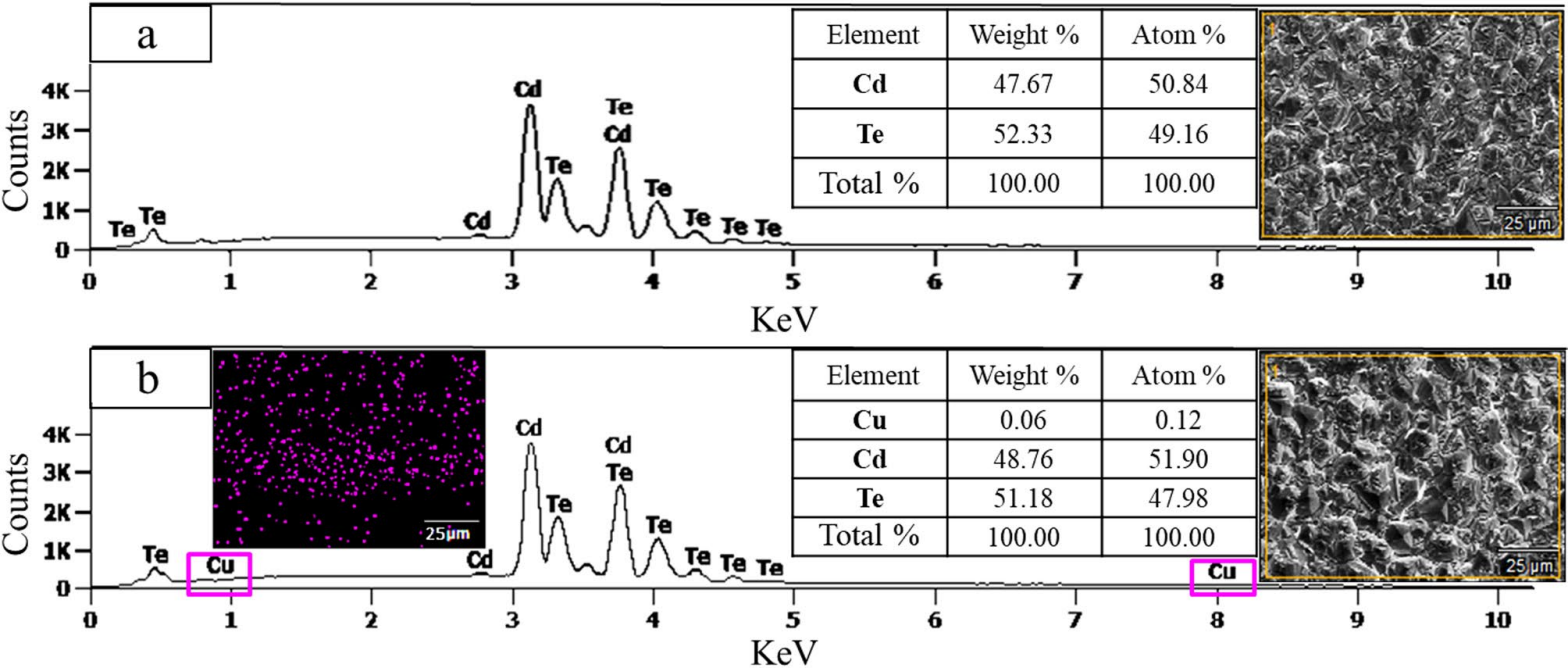
EDX results from the **a** Baseline and **b** optimally CuCl_2_ treated CdTe samples.

Adjusting the balance of negative and/or positive molarity in the treatment solution can somewhat restrain the formation of defects. CuCl_2_ treatment aims to minimize such recombination centers, ultimately enhancing the overall device operation. As depicted in Fig. 8, the treatment process has induced a slight shift in the atomic ratio of cadmium (Cd) and tellurium (Te), resulting in alterations in optoelectrical properties.

Since Te atoms are heavier, they contribute more to the overall mass of the sample despite having a lower atomic percentage, resulting in a p-type CdTe compound. Higher tellurium content in CdTe thin films has been explored across different studies, reporting Te as one of the most effective materials for reducing the potential energy barrier between CdTe and the metal back contact by increasing the hole concentration^51^. This strategic compositional adjustment in the elemental ratios serves as a key method for mitigating defect formation in various materials.

## Conclusion

This work conducts a systematic evaluation of the implications of CuCl₂ post-treatment on CdTe thin films deposited using the CSS technique, analysing the most suitable Cu inclusion strategy that enhances electrical performance while maintaining structural and morphological integrity. This study systematically investigates a broad range of CuCl₂ molarity on a logarithmic scale, establishing a definitive association among dopant concentration, defect chemistry, microstructural development, and charge transport behaviour in CdTe thin films.

Structural findings demonstrate that all treated films maintain the characteristic zinc-blende CdTe phase with a predominant (111) orientation, while no evidence of detectable Cu- or Cu–Te-based secondary phases throughout the examined range suggests that Cu incorporation occurs mainly via substitutional and defect-passivating mechanisms rather than through alloy formation. Nevertheless, a detailed comparison of concentrations indicates that Cu insertion is markedly non-linear: excessively low molarities (0.0005–0.001 M) facilitate superior crystallographic orientation and grain growth but inadequately activate Cu as an acceptor, leading to inadequate carrier concentration and elevated resistivity. Conversely, higher molarities (≥ 0.01 M) provoke excessive Cu diffusion, elevated microstrain, increased defect scattering, and deterioration of electrical property. At elevated CuCl₂ molarities, the decline in electrical performance—despite the augmented presence of Cu—is ascribed to a shift in the predominant Cu structure inside the CdTe lattice. Excess copper progressively accumulates at grain boundaries or fills interstitial and complicated defect states, functioning as compensatory centres and charged scattering sites instead of electrically active substitutional acceptors. This defect-driven self-compensation process restricts effective carrier activation and increases carrier dispersion, thereby diminishing nett carrier concentration and long-term electrical stability. These data affirm that Cu incorporation in CdTe is intrinsically non-linear and that electrical optimisation is dictated not by Cu concentration, but by regulated defect chemistry.

In the above scenario, the 0.005 M CuCl₂ condition consistently appears as a pivotal transition point across the examined datasets. Structurally, it signifies the initiation of regulated lattice alteration, as indicated by mild XRD peak displacements and FWHM widening, without the emergence of secondary phases or significant strain build-up. This concentration results in significant, well-coalesced grains with decreased grain-boundary density and enhanced compositional homogeneity, as verified by FESEM and EDX studies. The optical band gap of CdTe remained within its inherent value (~ 1.62 eV), suggesting no electronic distortion or degradation of absorber quality. notably, from an electrical perspective—essential for photovoltaic functionality—the 0.005 M treatment produces the highest carrier concentration, approximately 10^15^ cm^–3^, alongside the lowest resistivity and regulated mobility. The convergence of structural, optical, morphological, and electrical indicators confirms that 0.005 M is the ideal CuCl₂ molarity for efficient Cu activation in CdTe thin films.

Ultimately, the originality of this work resides in demonstrating CuCl₂ activation through a precise, experimentally validated equilibrium at which Cu acts primarily as an electrically active, defect-passivating dopant instead of a source of compensating defects or uncontrolled diffusion. The effective application of this optimised CuCl₂ treatment at an annealing temperature of 390 °C for 15 min presents a viable, eco-friendly alternative to traditional CdCl₂ activation methods, yielding superior carrier activation and enhanced absorber quality while maintaining material stability. The electrical characteristics directly influence CdTe absorber layer’s properties; hence, this study’s findings establish a solid foundation for the selective choices of dopant concentrations in CdTe photovoltaics and offer significant insights for the scalable production of high-performance CdTe solar cells. Future endeavours will concentrate on comprehensive diffusion-kinetics modelling, enhanced operational stability evaluations, and interface optimisation to effectively transfer these material-level advancements into device-level performance enhancements.

## Data Availability

The data that support the findings of this study are available within the article.
